# Environmental factors influencing the distribution of ammonifying and denitrifying bacteria and water qualities in 10 lakes and reservoirs of the Northeast, China

**DOI:** 10.1111/1751-7915.12260

**Published:** 2015-03-10

**Authors:** XinYu Zhao, Zimin Wei, Yue Zhao, Beidou Xi, Xueqin Wang, Taozhi Zhao, Xu Zhang, Yuquan Wei

**Affiliations:** 1 College of Water Sciences, Beijing Normal UniversityBeijing, 100875, China; 2 Life Science College, Northeast Agricultural UniversityHarbin, 150030, China; 3 State Key Laboratory of Environmental Criteria and Risk Assessment, Chinese Research Academy of Environmental SciencesBeijing, 100012, China

## Abstract

This study presents seasonal and spatial variations of the ammonifying bacteria (AB) and denitrifying bacteria (DNB) and physicochemical parameters in 10 lakes and reservoirs in the northeast of China. Water samples were collected in winter (January), spring (March), summer (July) and fall (November) in 2011. The study revealed that physicochemical parameters such as pH, dissolved oxygen (DO), NH_4_^+^-N and nitrate as nitrogen were closely related with the distribution of AB and DNB. Seasonally, the levels of AB presents gradually upward trend from winter to summer, and declines in fall and DNB were higher in spring and fall than summer and lowest in winter. Spatially, the annual average of AB among 10 lakes and reservoirs showed insignificant difference (*P* > 0.05), for DNB, Udalianchi and Lianhuan Lake were lower than others (*P* < 0.05). Regression correlation analysis showed that the levels of AB and DNB had a close relationship with nitrogen nutrition. Three principal components were identified of total variances which are conditionally classified by the ‘natural’ factor (PC1) and ‘nitrogen nutrients’ (PC2, PC3). According the principal component scores, cluster analysis detected two distinct groups: (C1) mainly affected by nitrogen nutrients and (C2) natural environmental factors.

## Introduction

Global nitrogen cycle (N-cycle) has increased attention since nitrogen loading have undoubtedly contributed to an increased occurrence of harmful in freshwaters, estuaries and coastal oceans (Herbert, 1999a). From a human point of view, the eutrophication of aquatic ecosystems by excess nitrogen has led to altered ecosystem function and structure, water quality degradation and economic loss (Bianchi *et al*., 1994). As a consequence of the high external loading with nitrate as nitrogen (NO_3_^−^-N) and nitrite as nitrogen (NO_2_^−^-N) growth of especially planktonic primary producers spring be enhanced, which can have profound effects on the quality of receiving waters.

On account of most plants and microorganisms in the water system couldn't make use of the nitrogenous organic matter directly, based on this, which should be transformed into absorbable components by microbial degradation. Degradation of organic matter leads to the formation of ammonia as nitrogen (NH_4_^+^-N) via ammoniation, which is either lost to the overlying water or oxidized to NO_3_^−^-N via nitrification at the oxic water interface (LeChevallier, 2003). Denitrification is a key process in the water nitrogen cycle since it decreases the amount of nitrogen available to the primary producers as the gaseous end-products (N_2_O and N_2_) diffuse into the atmosphere. Microbial communities in the aquatic ecosystems play a key role help in the nutrient recycling which involves nitrogen fixation, ammonification, nitrification and denitrification processes carried out by different microorganisms (Altmann, 2003). The ability to ammonify and denitrify is widely distributed among ammonifying and denitrifying bacteria (Payne, 1973; Herbert, 1982). These transformations are not only mediated by a metabolically diverse range of autotrophic and heterotrophic microorganisms but also strongly affected water quality and eutrophication control by the prevailing physicochemical conditions such as proper pH, dissolved oxygen (DO) and concentration of nitrogen nutrition (Davies *et al*., 1995; Juhna *et al*., 2007). In order to understand the influence factors of the nitrogen nutrition in water, we need to study the relationship between the environment factors and microorganism. In addition, a number of studies have shown in temperate aquatic ecosystems that microorganism showed distinct seasonal patterns governed principally by seasonal variation (Smith *et al*., 1985; Yoon and Benner, 1992).

Typical lakes and reservoirs in Northeast of China were important sources of drinking water areas. However, with the development of industry and agriculture in recent years, the water bodies of lakes and reservoirs had experienced water quality deterioration. As far as our knowledge is concerned, there are very few studies examining the dynamics of spatial-temporal variations of ammonifying bacteria (AB) and denitrifying bacteria (DNB), comparing the water quality among the typical lakes and reservoirs in the northeast of China. The present study's aim was to identify the distribution of the AB and DNB and the relationship with physicochemical characteristics in the water bodies. Then, 10 typical lakes and reservoirs were classified into groups with the similar levels of indicators which would be beneficial for the future management. The main objective was to provide basic information and scientific data for policy makers and for the researchers to deal with similar kinds of water system.

## Results and discussion

### Physicochemical parameters associated with distribution of ammonifying and denitrifying bacteria

**Fig 1:**
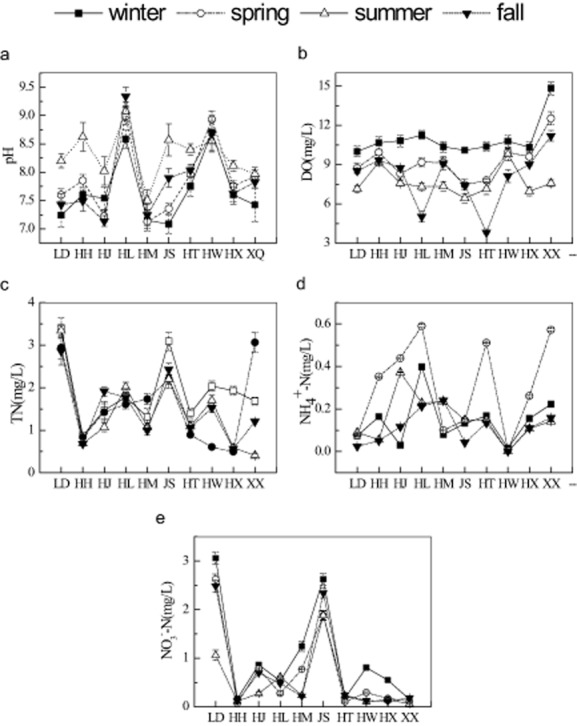
Seasonal changes of variables pH, DO, TN, NH_4_^+^-N and NO_3_^−^-N in 10 lakes and reservoirs.

The seasonal variations of water quality values and correlation coefficients to identity the interrelationships for 10 lakes and reservoirs are given in Fig. 1, pH is a significant indicator for the growth of AB and DNB (Venkatesharaju *et al*., 2010). Too much acid or alkaline pH would inhibit the growth of AB and DNB (Beversdorf *et al*., 2013). As shown in Fig. 1A, pH in 10 lakes and reservoirs showed slightly alkaline all year around, especially in summer which might be due to that water was cleaner in summer with lower total suspended solids (TSS; Table 1). There was significant negative correlation between pH and TSS (Table 1). Spatially, pH in Lianhuan Lake (HL) and Udalianchi (HW) were obviously more alkaline than other lakes throughout the year. HW is a volcano dammed lake which has been around lots of peralkaline rocks. For HL, slightly alkaline pH was preferable in water which was due to the high carbonate or bicarbonate (Table 1). Heavy metals could be removed by high carbonate and bicarbonate precipitates (Ahipathy and Puttaiah, 2006).

**Table 1:** Correlation matrix for levels of ammonia and denitrifying bacteria and physicochemical parameters in water samples

	TN	NH_4_^+^-N	LgAB	NO_3_^−^-N	NO_2_^−^-N	LgDNB	pH	DO	TSS	EC	TA	COD_Mn_	HCO_3_^−^
NH_4_^+^-N	0.034												
LgAB	−0.145	0.871**											
NO_3_^−^-N	0.904**	−0.202	−0.418										
NO_2_^−^-N	0.789**	−0.287	−0.236	0.735*									
LgDNB	0.421	−0.121	−0.263	0.775*	0.293								
pH	−0.178	0.616	0.639	−0.368	−0.255	−0.090							
DO	−0.239	0.807*	0.304	−0.785*	−0.340	−0.744*	−0.214						
TSS	0.460	−0.141	−0.189	0.488	0.666*	0.233	−0.424	−0.161					
EC	0.077	0.466	0.523	−0.144	−0.165	0.127	0.805**	−0.324	−0.574				
TA	0.005	0.463	0.526	−0.204	−0.226	0.127	0.822**	−0.344	−0.577	0.995**			
COD^Mn^	−0.458	0.799**	0.512	−0.646^*^	−0.629	−0.210	0.675*	0.247	−0.517	0.523	0.546		
HCO_3_^−^	−0.002	0.472	0.536	−0.217	−0.231	0.100	0.831**	−0.324	−0.583	0.995**	1.000**	0.556	
CO_3_^2−^	0.073	0.421	0.468	−0.119	−0.187	0.284	0.733*	−0.436	−0.530	0.976**	0.981**	0.493	0.975**

* means significant difference (p < 0.05)

** means significant difference (p < 0.01).

DO was another important indicator to affect the process of microbial metabolism (Ahipathy and Puttaiah, 2006). Figure 1B also showed seasonal and spatial variation of DO for 10 lakes. Temporally, DO was the highest in winter and the lowest in summer. This is due to the breeding growth of microorganisms during summer, consumed considerable amount of oxygen with the decomposition of organic matter. Spatially, lower DO levels were detected at HL and Taoshan Reservoir (HT) in fall, and the highest annual averages of DO were observed in Xingkai Lake (XQ), which were probably due to the bigger wind and waves.

One of the most relevant factors to affect the distribution of AB and DNB is eutrophication. Total nitrogen (TN), NH_4_^+^-N and NO_3_^−^-N are the main nitrogen nutrients in the water body (Fig. 1C–E). As spring is the cultivation time along the river, a rapid release of ammonium to the water body, NH_4_^+^-N was the highest in March. Variations of NO_3_^−^-N were similar to those of TN, and there was a positive correlation between each other (Table 1). Spatial difference was most obviously reflected in Dahuofang (LD) and Songhua Lake (JS) with significantly high levels of TN and NO_3_^−^-N. The accumulation of NO_3_^−^-N in LD) and Songhua Lake (JS) showed that the nitrogen removal capacity by denitrification was stronger than others (Herbert, 1999a). In contrast, extremely low NO_3_^−^-N stimulated the level of ammonification at the expense of the denitrification in the water (King and Nedwell, 1985).

### Seasonal and changes in distribution of ammonifying and denitrifying bacteria

**Fig 2:**
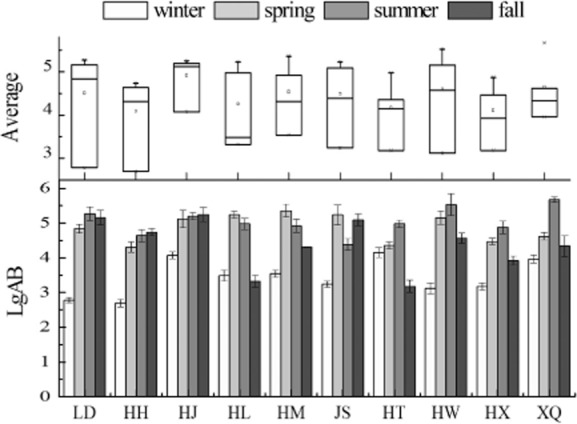
Levels of AB in 10 water reservoirs. Each datum represents the mean ± standard deviation. The average of AB among the 10 reservoirs is not significantly different (*P* > 0.05) according to one-way ANOVA.

The amount of ammonifying bacteria (AB) presents gradually upward trend from winter to summer, and declines in fall were found in almost 10 lakes (Fig. 2). Extremely low temperature in winter goes against the growth of AB, and a marked increase of AB in spring followed a rapid release of ammonium to the water body (Donnelly and Herbert, 1996; Poulin *et al*., 2007). The upward trend is still preserved in summer, due to the more frequently rainstorms and the rise of temperature in summer, water flow rates speed up which lead to much organic matter contain nitrogen flowed into the surface water as the substrates for AB (George *et al*., 2004; Djuikom *et al*., 2006). There were significant positive correlations between NH_4_^+^-N, DO and AB (Table 1). It is indicated that high values of NH_4_^+^-N may have stimulated the growth rate of AB, and ammoniation process also needs oxygen existing in the water body (Yang *et al*., 2007). Spatially, one-way analysis of variance (ANOVA) showed that the annual average of AB among 10 lakes and reservoirs showed insignificant difference (*P* > 0.05), while lower levels of AB were found in HL and HT in fall. Very low DO is a disadvantage of AB overgrowth of HT and HL (below 6 mg l^−1^) (Fig. 1A).

**Fig 3:**
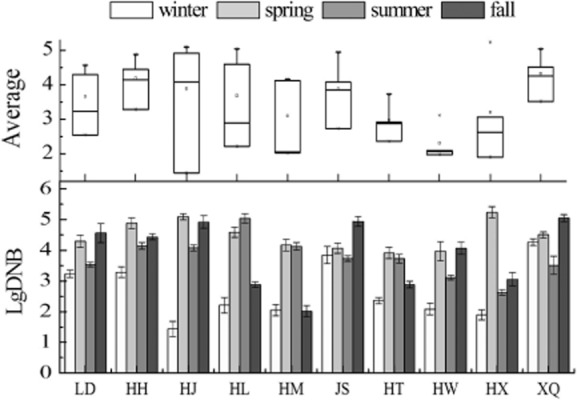
Levels of DNB in 10 water reservoirs. Each datum represents the mean ± standard deviation. The average of DNB in HW, HL, HT is significantly different than other reservoirs (*P* < 0.05) according to one-way ANOVA.

Seasonal changes have a large impact on the distribution of denitrifying bacteria (Fig. 3). There was significant negative correlation with DO and DNB (Table 1). Lowest numbers of DNB were recorded when DO is at maximum during winter. This coincided with that nitrate can be reduced to N_2_O by a number of fermentative anaerobe bacteria, and excessive DO concentrations inhibited the growth of DNB (Dunn *et al*., 1980; MacFarlane and Herbert, 1982; Keith and Herbert, 1983). Generally, the numbers of DNB were higher in spring and fall than in summer. This is in accordance with the study that the capacity for NO_3_^−^-N reduction to NH_4_^+^-N was higher than reduction to N_2_ which leads to the capacity for denitrification, which has been lower in summer (King and Nedwell, 1985). Spatially, ANOVA showed that the annual averages of denitrifying bacteria in HW and HL are lower than other lakes (*P* < 0.05). For these two lakes, lower levels of DNB were resulted in the alkaline pH value of water which is adverse to DNB growth (Fig. 1). It was worth mentioning that extremely low DO was also limited to DNB growth in HT (below 4 mg l^−1^) in fall (Fig. 1).

### Nitrogen compounds associated with distribution of ammonifying and denitrifying bacteria

**Fig 4:**
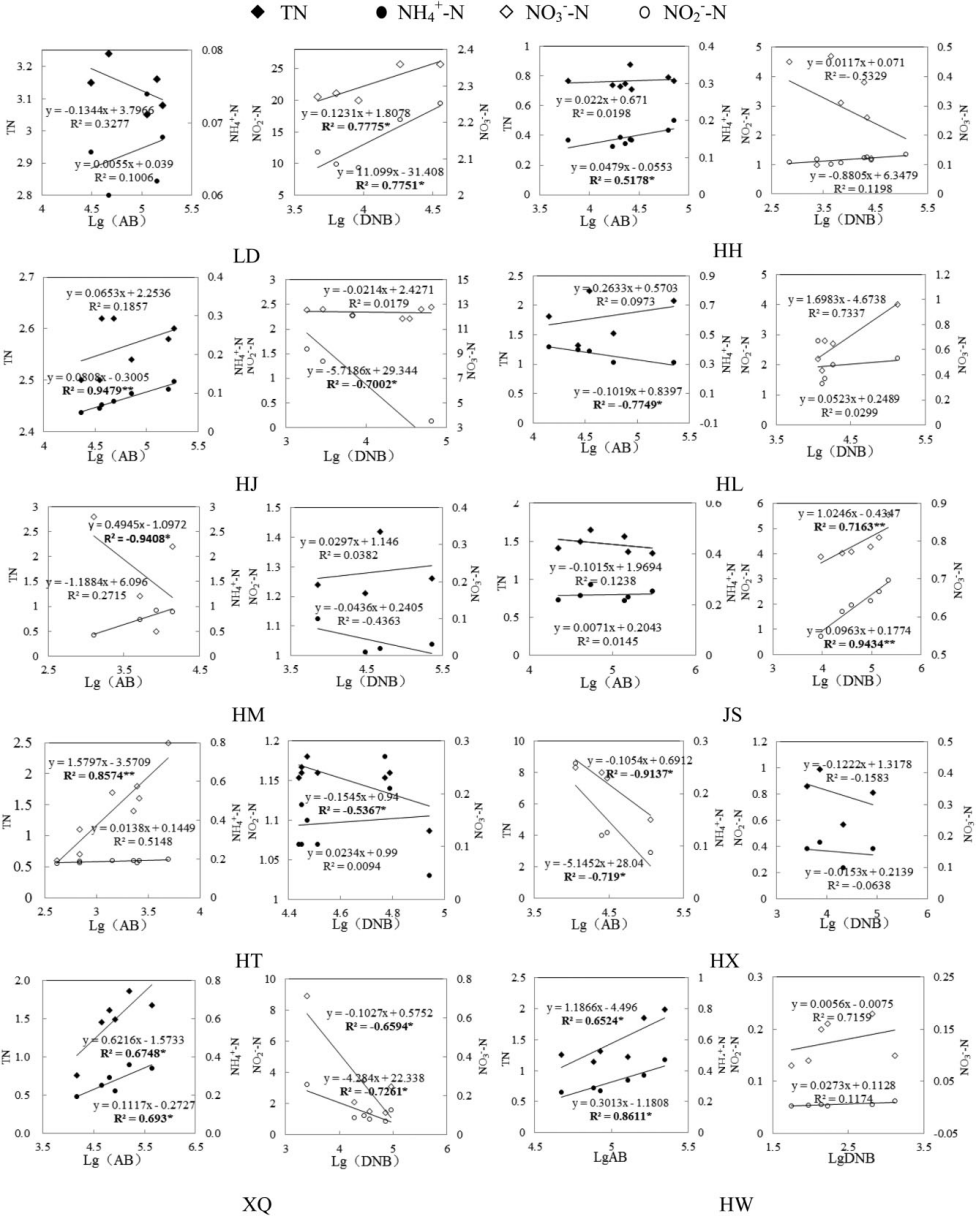
Regression correlation matrix for AB and DNB levels and nitrogen compounds in 10 lakes and reservoirs.

It is clearly evident from the foregoing section that ammoniation and denitrification in water system were subject to a complex array of regulatory mechanisms involving both physicochemical and biological factors (Herbert, 1999a). Hence, there is a need to understand the relationship between the average microbial biomass of AB, DNB and nitrogen nutrients for each lake by regression correlation analysis (Fig. 4). Significant correlation with the amount of LgAB and TN or NH_4_^+^-N were found in Hongqipao (HH), Jingbo Lake (HJ), HL, HW, Xingkai Lake (XQ). Strong association were found between LgDNB and NO_3_^−^-N or NO_2_^−^-N in LD, HJ, JS, HT and XQ, while it should be noted that positive correlation only found in LD and JS, indicating that excessive NO_3_^−^-N and NO_2_^−^-N would stimulate the growth of DNB, denitrification would be the dominant process in LD and JS (King and Nedwell, 1985).

### Classification for lakes and reservoirs

In order to explain variance of large dataset of the related indicators with small groups, principal components analysis (PCA) was a conducted pattern recognition technique (Hopke, 1985). Three principal components were investigated with eigen-values > 1 summing almost 85.6% of the total variance in the water dataset (Table 2). The first PC (natural factor; PC1), accounting for 48.618% of the total variance was correlated with total alkalinity (TA), carbonate (CO_3_^2−^), electrical conductivity (EC), bicarbonate (HCO_3_^−^) and pH. This ‘natural’ factor was represented internal environmental characteristic factors of water's natural quality. These factors were mainly influenced from non-point sources such as fields, base erosions, soil erosion and atmosphere deposition (Kannel *et al*., 2007). Nitrogen nutrients PC2 and PC3, accounting for 22.749% and 14.219% of the total variance, respectively, can be grouped as nutrients. PC2 were correlated primarily with TN, NO_3_^−^-N, NO_2_^−^-N, LgDNB and secondarily with TSS and chemical oxygen demand (COD_Mn_). The sources of these variables were mainly from anthropogenic pollution, such as municipal solid waste. PC3 were primary correlated with LgAB, DO and NH_4_^+^-N. Degradation of organic matter leads to the formation of NH_4_^+^-N based upon the oxic conditions of the water environment (Altmann *et al*., 2003).

**Table 2:** Rotated (varimax rotation) factor loadings and communalities

	PC1	PC2	PC3
TA	0.978	−0.065	0.110
CO_3_^2−^	0.977	0.022	0.047
EC	0.975	−0.007	0.130
HCO_3_^−^	0.974	−0.078	0.123
pH	0.802	−0.243	0.302
TN	0.061	0.961	0.108
NO_3_^−^-N	−0.101	0.937	−0.170
NO_2_^−^-N	−0.202	0.851	0.031
LgDNB	0.140	0.689	−0.386
TSS	−0.562	0.590	0.170
COD_Mn_	0.520	−0.572	0.505
LgAB	0.433	−0.228	0.843
NH_4_^+^-N	0.436	−0.099	0.795
DO	−0.451	0.251	0.677
Percent variance (%)	48.618	22.749	14.219

**Fig 5:**
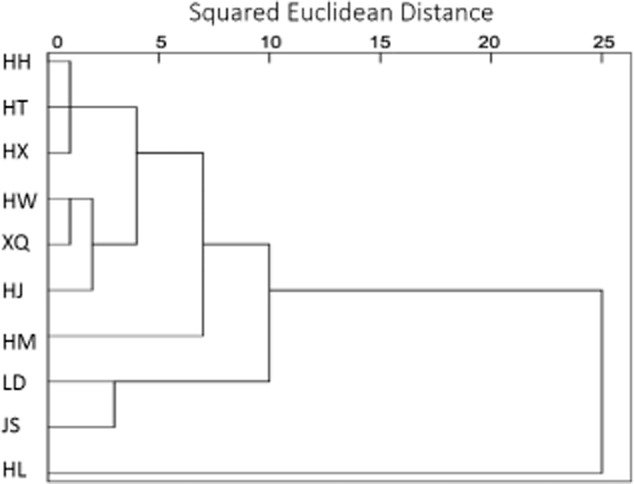
Dendrogram with Baverage's linkage and correlation distance obtained from hierarchical cluster analysis for 10 water reservoirs.

Cluster analysis (CA) was applied to reveal a dendrogram in the 10 lakes and reservoirs (Fig. 5). After determining the number and identity of possible sources affecting surface waters by using PCA, site similarity were calculated next by CA on the principal component scores. It is possible to classify 10 lakes and reservoirs among various source components obtained by PCA which are grouped into distinct pattern of two main clusters: Cluster 1 (C1) composed of nine stations has two sub clusters: C1(a) consists of seven lakes [HH, HT, Xiquanyan (HX), HW, XQ, HJ and Mopanshan (HM)], which are main contributors to PC3. C1(b) consists of JS, LD, which appeared to be related to PC2. Cluster 2 only includes HL, which was mostly related to PC1, second with PC2 and PC3.

Cluster 1(a) showed significant relation with NH_4_^−^-N, DO and AB, accounting for high concentration organic matter, and NH_4_^−^-N was dominant compared with the other dissolved forms of nitrogen, ammonification was stronger in HH, HT, HX, HW, XQ, HJ and HM. Most of these lakes were aquacultures which were adjacent to land or residential areas; the source of NH_4_^−^-N might be associated with nitrogen compounds used in agriculture runoffs or human waste (Wakida and Lerner, 2005). C1 (b) showed that denitrification was a major role in LD and JS. This contributed to the higher level of NO_3_^−^ -N compared with other dissolved forms of nitrogen in these areas. Bianchi and colleagues (1994) demonstrated that denitrification occurs in highly turbid estuarine waters with a high nitrate concentration (Bianchi *et al*., 1994). As the nitrate concentration increased, denitrification became the dominant process (King and Nedwell, 1987). Therefore, typical lake and reservoirs of northeast China are natural highly productive environments which in recent years have been subject to increased anthropogenic inputs of nitrogen arising from such diverse sources such as fertilizer run-off, selvage discharges or aquaculture (Ho *et al*., 2003). Cluster 2 corresponded to TA, CO_3_^2−^, EC, HCO_3_^−^ and pH, composed only HL. Clearly, nitrogen nutrient was not the major pollution in HL, and was associated with the base erosions, soil erosion and atmosphere deposition (Kannel *et al*., 2007).

## Experimental procedures

### Field sampling

**Fig 6:**
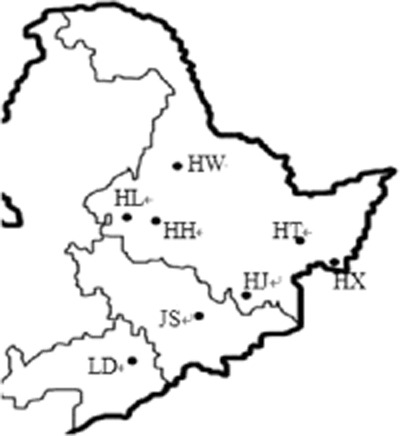
Distribution of 10 water reservoirs.

Water sampling was conducted in winter (January), spring (March), summer (June) and fall (September) in 2011. Ten water storage reservoirs which were located in three provinces of northeast China were selected. They were independent from each other (Fig. 6). These include Heilongjiang Province, Udalianchi, Xingkai Lake, Jingbo Lake, Mopanshan, Hongqipao, Lianhuan Lake, Taoshan Reservoir, Xiquanyan, Jilin province, Songhua Lake, Liaoning province, Dahuofang.

### Determination of denitrifying and ammonifying bacteria

Ammonifying bacteria was cultivated in peptone ammoniation medium, and DNB was in denitrifying bacteria culture medium (Rodina, 1972). Numbers of AB and DNB in water samples was conducted using most probable number.

### Analysis of physicochemical parameters

Water samples were collected from approximately 16 m below the surface with an open-mouthed bottle (for analysis of physicochemical parameters) and a sterile 1000 ml glass vessel (for bacteria analyses). For each reservoir, different numbers of sampling points were selected according to the location or shape, and all of the samplings were conducted in 9:30–11:30 (a.m.). In this study, 12 parameters were detected, which were pH, EC, DO, TN, NO_3_^−^-N, NO_2_^−^-N, NH_4_^+^-N, COD_Mn_, TA, HCO_3_^−^, CO_3_^2−^, TSS. The data quality was checked by careful standardization, procedural blank measurements, spiked and duplicate samples. The analysis methods were based on standard methods in water and wastewater monitoring analysis method (4th edition). Part of the data were shown in Fig. 1A–E, and all data processing used Origin (8.0).

### Data analysis

One-way analysis of variance followed by the LSD comparisons test were used to compare AB and DNB levels among the reservoirs in different seasons respectively. Pearson linear correlations and PCA were used to study the relationship between the annual mean of AB, DNB and physicochemical parameters. Regression correlation matrix was used to identify the correlation between levels of AB and DNB and nitrogen compounds in each lake and reservoir. In PCA analysis, factors were denitrified via varimax rotation with eigenvalue > 1. Cluster analysis was calculated by principal component scores. It makes it possible to classify water stations among various source components obtained by PCA. The Baverage's linkage cluster method was applied to the data. All data processing used spss (19.0).

## Conclusions

This study indicated that seasonal variation and physicochemical properties of the water would influence the levels of AB and DNB in directly and indirectly way. Statistical analysis demonstrated that AB and DNB were closely related to physicochemical factors such as pH, DO, NH_4_^+^-N and NO_3_^−^-N. Regression correlation analysis showed that AB and DNB were closely related to nitrogen-related indictors and strong positive correlation between DNB and NO_3_^−^-N, NO_2_^−^-N were only found LD and JS. Principle component analysis revealed that the major factors in 10 lakes and reservoirs were: natural factors (PC1: TA, CO_3_^2−^, EC, HCO_3_^−^, pH) and nitrogen nutrients (PC2: TN, NO_3_^−^-N, NO_2_^−^, LgDNB; PC3: LgAB, DO, NH_4_^+^-N). The cluster analysis detected two distinct groups: (a) C1 (HH, HT, HX, HW, XQ, HJ and HM) was major affected by NH_4_^+^-N, AB, and ammoniation was stronger; (b) (LD and JS) was mainly affected by the TN, NO_3_^−^-N, NO_2_^−^-N and DNB, denitrification was a major role; C2 (HL) was majorly affected by natural factors.

## Conflict of interest

None declared.
